# Training of spatial cognitive abilities reduces symptoms of visually induced motion sickness

**DOI:** 10.3389/fpsyg.2024.1415552

**Published:** 2024-09-02

**Authors:** Fan Wang, Shuai Pan, Xiao-wen Li, Jia-mei Lu, Chuan-jing Qiu, Meng-hang Jiang, Zhan-guo Jin, Sheng-guang Yan

**Affiliations:** ^1^School of Psychology and Mental Health, North China University of Science and Technology, Tangshan, China; ^2^School of Public Health, North China University of Science and Technology, Tangshan, China; ^3^Xinghua City People’s Hospital, Taizhou, China; ^4^College of Science, North China University of Science and Technology, Tangshan, China; ^5^Air Force Medical Center, PLA, Beijing, China; ^6^Hebei Key Laboratory of Occupational Health and Safety for Coal Industry, Tangshan, China

**Keywords:** virtual reality, visually induced motion sickness, spatial cognition ability, mental rotation test, intervention

## Abstract

**Purpose:**

This study aims to explore the effectiveness of enhancing individual spatial cognitive abilities in alleviating the negative symptoms of visually induced motion sickness (VIMS). Additionally, it seeks to develop innovative intervention methods to improve spatial cognition and identify new treatment approaches for VIMS.

**Methods:**

The study investigated the impact of innovative interventions on spatial cognitive abilities and their modulation of VIMS susceptibility. A total of 43 participants were recruited (23 in the experimental group and 20 in the control group). The experimental group underwent six sessions of spatial cognitive ability training, while the control group engaged in activities unrelated to spatial cognition.

**Results:**

The analysis revealed that the spatial cognitive ability scores of the experimental group significantly improved after the intervention. Furthermore, the experimental group exhibited significant differences in nausea, oculomotor, disorientation, and total SSQ scores before and after the intervention, indicating that the intervention effectively mitigated VIMS symptoms.

**Conclusion:**

This study developed a virtual reality training method that effectively enhances individual spatial cognitive abilities and significantly alleviates VIMS symptoms, providing a novel and effective approach for VIMS intervention and treatment.

## 1 Introduction

### 1.1 Visually induced motion sickness

Virtual reality (VR) technology has gained widespread popularity due to its ability to create highly immersive and realistic digital environments, engaging users through both visual and auditory stimuli (Yu and Zhou, 2017). However, similar to the motion sickness experienced during car or airplane travel, VR technology can also induce motion sickness in susceptible individuals. Symptoms of visually induced motion sickness (VIMS) include headaches, nausea, vomiting, drowsiness, and persistent fatigue, particularly with prolonged use or intense stimulation (Solimini, 2013; Cha et al., 2021).

This VR-induced motion sickness is known as VIMS, also referred to as simulator sickness (SS) or simulator adaptation syndrome (SAS), which is a subtype of motion sickness (Cha et al., 2021). The increasing reliance on electronic devices in modern society has elevated the risk of VIMS, making the prevention and alleviation of VIMS a crucial issue for the future development of VR technology (Cha et al., 2021; Tan et al., 2021).

### 1.2 Pathogenesis

#### 1.2.1 Sensory conflict theory

The Sensory Conflict Theory, proposed by Reason and colleagues, posits that multiple sensory organs (such as the visual system, vestibular system, and proprioceptive system) must work in harmony to provide consistent information to the brain during motion. When these sensory inputs are inconsistent or conflicting, the brain receives discordant information, leading to the occurrence of VIMS (Oman, 1990). Therefore, enhancing spatial cognitive abilities can help individuals better coordinate and integrate these sensory inputs, thereby reducing sensory conflicts.

#### 1.2.2 Postural instability theory

The Postural Instability Theory suggests that postural instability is the minimal state of uncontrolled motion within the perception-action system. When individuals enter a new motion environment, they need to adjust their posture to adapt to the new conditions. If the posture does not match the environment, it may trigger VIMS (Feigl et al., 2019). Improving spatial cognitive abilities can help individuals adapt more quickly to new environments, thus reducing the risk of VIMS caused by postural instability.

#### 1.2.3 Eye movement theory

The Eye Movement Theory, introduced by Ebenholtz, suggests that VIMS arises from optokinetic nystagmus and vestibular-ocular reflexes. These eye movements lead to tension in the eyes and surrounding muscles, causing symptoms such as headaches, difficulty concentrating, and eye fatigue (Ebenholtz, 1992). Training spatial cognitive abilities through VR equipment can improve eye movement control, thereby reducing the occurrence of VIMS.

### 1.3 Treatment methods for VIMS

The treatment methods for VIMS typically include medication or vestibular adaptation training. In recent years, psychological therapies have gained significant attention in the treatment of VIMS (Hui et al., 2022). Li and Xu (2011) found that psychological variables such as anxiety or depression are closely related to the occurrence of VIMS, and psychological training can effectively alleviate VIMS. Additionally, Cowings et al. (2018) suggested that biofeedback training shows better efficacy and fewer side effects compared to medication. Smyth et al. (2021) demonstrated through experiments that training spatial cognitive abilities is also an effective non-pharmacological intervention, which can mitigate VIMS symptoms by enhancing spatial cognition.

Moreover, Chu et al. (2012) and Seung et al. (2021) employed non-invasive Transcutaneous Electrical Nerve Stimulation (TENS) therapy to treat motion sickness. This method, based on traditional Chinese medicine principles, stimulates acupoints such as Neiguan and Zusanli. Experimental evidence has shown that this approach effectively alleviates motion sickness by increasing sympathetic nerve activity and reducing parasympathetic activity, thereby alleviating discomfort and cognitive impairments.

Since the primary cause of VIMS is the sensory mismatch between visual and vestibular motion cues, Park et al. (2023) proposed that distraction methods can reduce VIMS in VR environments by guiding users to focus on the areas of the screen with the least movement, thereby alleviating discomfort symptoms.

### 1.4 Spatial cognitive abilities

Spatial cognitive ability refers to an individual’s capacity to understand and remember the spatial relationships between objects. Visual–spatial representation is the core of spatial ability and a crucial factor in perceiving and interacting with the environment (Zhang et al., 2023). Consequently, the Mental Rotation Test (MRT) is an effective tool for assessing spatial cognitive abilities and is widely used in the field of cognitive neuroscience (Langlois et al., 2020). Additionally, Tada et al. (2024) demonstrated that virtual reality technology can also be used to measure spatial cognitive abilities.

### 1.5 Training spatial cognitive abilities

Traditional methods for training spatial cognitive abilities have typically involved pen-and-paper techniques. However, the application of electronic devices and VR technology has become increasingly prevalent in recent years. Research has shown that video games and computer-aided design (CAD) programs can effectively enhance visual–spatial abilities (Spence and Feng, 2010; Tseng and Yang, 2011). Slatenšek et al. (2021) argues that virtual reality technology allows users to interact with training stimuli, providing a superior training experience. Additionally, VR devices have been proven to be effective intervention tools for improving conditions related to vestibular dysfunction and enhancing spatial cognitive abilities (Pavlou et al., 2012; Tefertiller et al., 2019; Vleet et al., 2020).

In this study, VR devices were used to induce VIMS in susceptible individuals, and a spatial cognitive assessment system developed by the Dizziness Diagnosis and Treatment Research Center was employed to systematically evaluate and train participants’ spatial cognitive abilities. The objective of this study is to assess the effectiveness of spatial cognitive ability training in improving the cognitive levels of highly VIMS-susceptible individuals and to evaluate its role in alleviating VIMS symptoms. This will provide a basis for developing more accurate VIMS treatment strategies.

## 2 Materials and methods

### 2.1 Participants

#### 2.1.1 Inclusion criteria

Inclusion criteria were: no ear canal deformities, external ear infections, tympanic membrane perforation, or inner ear diseases; corrected or uncorrected vision of at least 4.8; no history of mental disorders, hypertension, cerebrovascular occlusion, coronary heart disease, asthma, epilepsy, or substance abuse; ability to undergo vestibular function tests confirming no abnormalities; and no consumption of any psychotropic drugs or alcohol within 48 h prior to the experiment.

#### 2.1.2 Ethic

This study was approved by the Ethics Review Committee of the PLA Air Force Medical Center [Air Force Special (Scientific Research) no. 2022-203-PJ01]. All participants voluntarily took part in the study, signed informed consent forms, and were compensated upon completing the experiment.

### 2.2 Experimental design

A systematic sampling method was utilized to recruit and select 97 university students for this study. VIMS was induced in participants, followed by the completion of the Simulator Sickness Questionnaire (SSQ). Participants scoring above 50, indicative of high susceptibility, were selected, resulting in a final sample of 46 students. Initial SSQ scores were recorded to establish a baseline for subsequent interventions.

Participants were then randomly assigned to either the experimental group or the control group. The experimental group underwent six sessions of spatial cognitive ability training, while the control group engaged in activities unrelated to spatial cognition, such as reading or self-study. To ensure internal validity, the time and location of the interventions were kept consistent across both groups to minimize experimental errors.

After three sessions of intervention, all participants completed the MRT to assess the short-term effects of the intervention on spatial cognitive abilities.

### 2.3 Equipment and materials

#### 2.3.1 Experimental equipment

This study utilized HTC VIVE VR equipment, including a VR headset and two handheld controllers. The VR headset features a single-eye resolution of 1440×1600 and a combined dual-eye resolution of 2880×1600, with a refresh rate of 90 Hz. The headset is ergonomically designed with adjustable lenses, interpupillary distance, and headset and headphones, ensuring a comfortable user experience. The handheld controllers are equipped with photodetectors that capture the direction and position of the headset, enabling precise control within the virtual environment. A high-performance computer with a 24-inch Philips monitor and the Steam VR plugin was used during the experiments. The spatial cognitive ability assessment system, developed by the Air Force Medical Center, is based on the Unity3D engine and includes programs for inducing visually induced motion sickness as well as testing and training spatial cognitive abilities.

#### 2.3.2 Experimental materials

This study aims to address the following two questions:

1. Is there a statistically significant difference in spatial cognitive abilities (MRT scores) before and after the intervention?
2. Is there a statistically significant difference in SSQ scores before and after the intervention?

**Figure 1:**
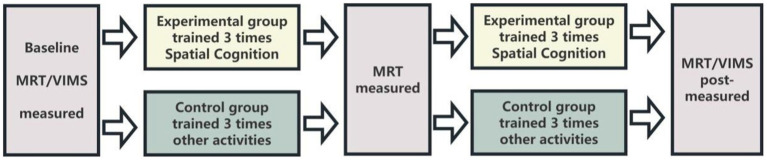
Experimental flowchart.

The specific experimental procedure is as follows (Figure 1):

##### 2.3.2.1 Spatial cognitive ability test

**Figure 2:**
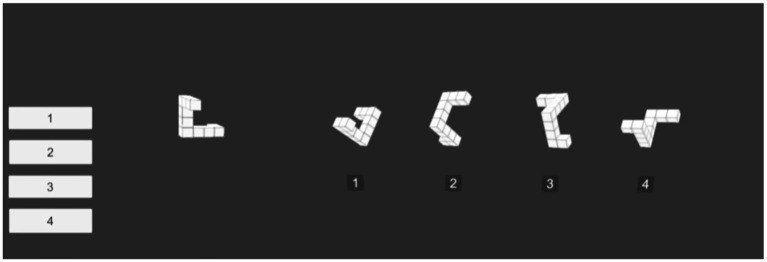
Visual spatial cognitive ability test in VR environment.

The MRT is a commonly used tool for assessing spatial cognitive abilities. This study utilized a VR version of the MRT, where each question consists of one original stimulus and four similar stimuli, derived by rotating the original version around the z-axis at different angles (30°, 60°, 90°, 120°, 150°, 180°) to create mirror versions. A total of 96 types of stimuli were used. During the training phase, questions randomly presented three-dimensional target figures and four possible matching stimuli, requiring participants to identify the two matching rotated figures within 15 s. The formal testing phase included 24 questions, with each correct answer worth 1 point, making the maximum score 24 points. Researchers recorded the number of correct answers on a computer (Figure 2).

##### 2.3.2.2 VIMS stimulation and assessment

**Figure 3:**
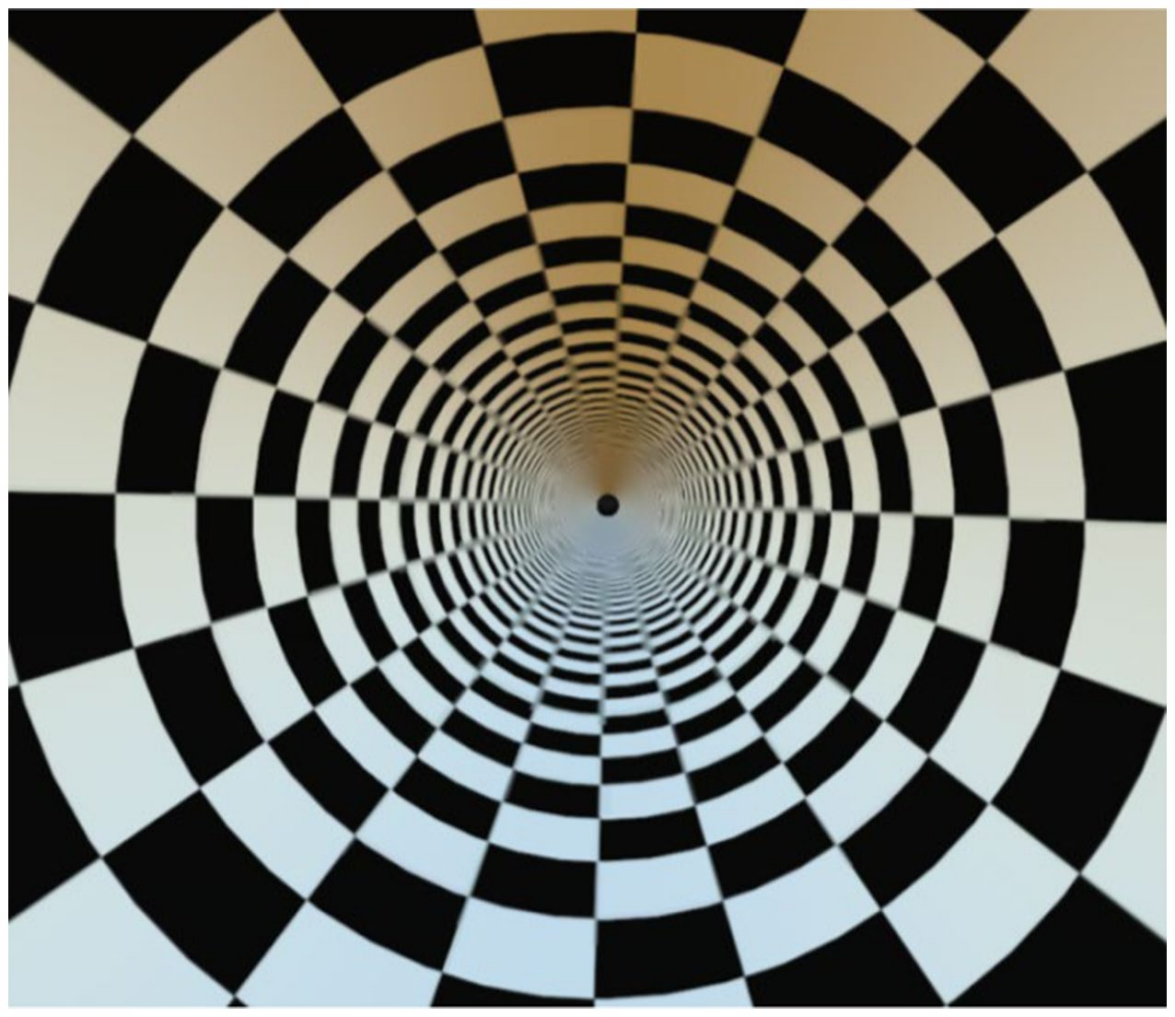
Visualization of VIMS induction.

To induce VIMS, a black and white checkerboard pattern was rotated counterclockwise at a speed of 60°/s in the VR environment, both before and after the intervention. Participants were required to continuously view the checkerboard for 15 min, then rest for 15 min before completing the SSQ (Figure 3).

The SSQ is a critical tool for assessing VIMS (Kennedy et al., 1993). It includes three subscales: Disorientation (e.g., difficulty concentrating), Nausea (e.g., general discomfort), and Oculomotor symptoms (e.g., eye strain, focusing issues), comprising a total of 16 items. Participants rated each item on a 4-point scale from 0 to 3. The total SSQ score and subscale scores were calculated using a specific formula to evaluate the severity of VIMS.

##### 2.3.2.3 Spatial cognitive ability training

In the virtual scene, the original stimulus was positioned on the left side, while the comparison stimulus was on the right side. During the test, if the right-side stimulus matched the left-side one (differing only in rotation), participants pressed the trigger button on the VR controller with their right hand (indicating “yes”); if it was a mirror image, they pressed the trigger button with their left hand (indicating “no”). Participants were required to make quick and accurate judgments.

**Figure 4:**
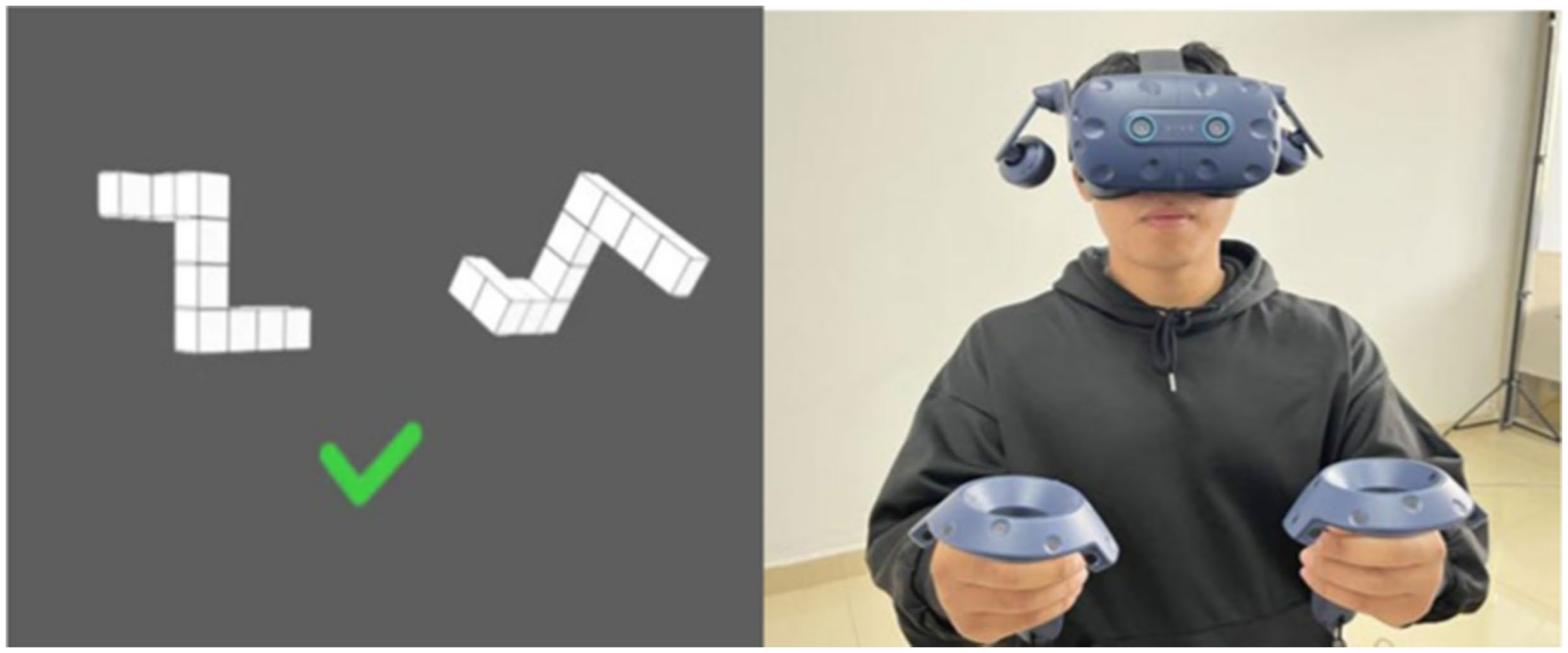
Spatial cognitive ability training diagram.

The training included two versions, each consisting of 48 and 24 questions respectively, with half being congruent (24 and 12 questions) and the other half incongruent (24 and 12 questions). The questions were presented randomly and rotated around the z-axis, requiring participants to judge as quickly and accurately as possible (Figure 4).

### 2.4 Data analysis

Statistical analyses were performed using SPSS version 26.0. Repeated measures analysis of variance (ANOVA) was employed to analyze the MRT scores before the intervention, after three interventions, and after six interventions. Paired samples t-tests were used to analyze the SSQ scores. The significance level for all tests was set at α = 0.05, and two-tailed tests were applied.

Data analysis and graphical representation were performed using SPSS version 26.0 software and Origin 2022 (OriginLab Corporation, United States).

## 3 Results

### 3.1 Participant attrition in the experiment

All participants were randomly assigned to either the experimental group (*n* = 23) or the control group (*n* = 20). In the control group, three participants voluntarily withdrew due to personal reasons and did not attend the scheduled tests.

### 3.2 General demographic characteristics of participants

The experimental group consisted of 23 participants, including 10 males (43.5%) and 13 females (56.5%). The control group initially consisted of 20 participants (with 3 later withdrawing), including 7 males (35%) and 13 females (65%). All participants signed informed consent forms before the experiment and received compensation upon completion. Baseline comparisons of age and gender between the experimental and control groups showed no statistically significant differences (*p* > 0.05), indicating the comparability of the two groups.

### 3.3 Main effects of spatial cognitive ability levels

To analyze the MRT scores of participants at different time points, Mauchly’s test of sphericity was conducted to assess the equality of the variance–covariance matrix of spatial cognitive abilities. The results indicated that the assumption of sphericity was not violated (χ^2^ = 4.33, *p* = 0.11), suggesting that the MRT scores of the experimental and control groups at different time points had equal variance–covariance matrices. Consequently, the Greenhouse–Geisser correction was not necessary, affirming the reliability of the analysis results.

**Figure 5:**
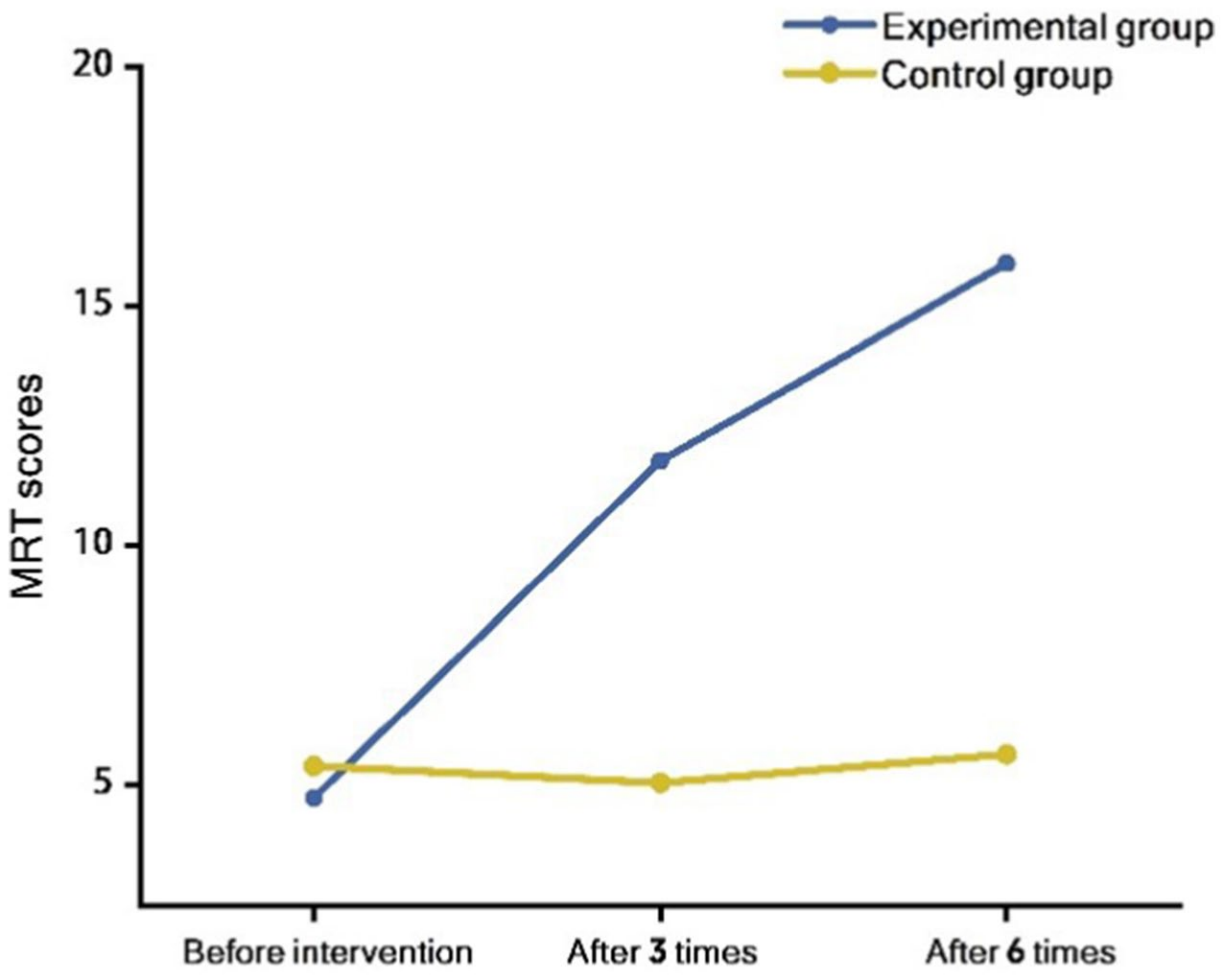
Interaction effect of time and group on MRT scores: comparison of experimental and control groups at different time points.

A repeated measures ANOVA was conducted on the MRT scores of participants in the experimental and control groups before training, after 3 training sessions, and after 6 training sessions. The analysis revealed a significant time effect (*F* = 36.62, *p* < 0.001), indicating that the spatial cognitive levels of both groups changed over time during the intervention. The between-group effect was also statistically significant (*F* = 47.39, *p* < 0.001), demonstrating differences in spatial cognitive levels between the experimental and control groups. Additionally, the interaction effect between the two groups’ spatial cognitive levels was statistically significant (*F* = 34.54, *p* < 0.001), indicating that different intervention methods had varying impacts on the spatial cognitive levels of the two groups (Figure 5).

### 3.4 Individual effect testing of spatial cognitive levels

Experimental data indicated that the MRT scores of the experimental group increased from 4.74 ± 2.12 before the intervention to 11.78 ± 5.01 after three interventions, and further to 15.91 ± 4.86 after six interventions. In contrast, the control group’s MRT scores slightly decreased from 5.40 ± 2.68 before the intervention to 5.05 ± 2.52 after three interventions, and only marginally increased to 5.65 ± 3.12 after six interventions.

A one-way repeated measures ANOVA revealed a significant improvement in spatial cognitive levels for the experimental group post-intervention compared to pre-intervention (*F* = 53.64, *p* < 0.001), whereas no statistically significant change was observed in the control group (*F* = 0.37, *p* = 0.69). After applying the Bonferroni correction, the differences in scores for the experimental group before the intervention, after three interventions, and after six interventions remained statistically significant (*p* < 0.0167), while the changes in the control group’s scores were not significant at any time point (*p* > 0.0167).

Further factor analysis results indicated no significant difference in spatial cognitive levels between the experimental and control groups before the intervention (*F* = 0.81, *p* = 0.37). However, significant differences were observed after three interventions (*F* = 29.55, *p* < 0.001) and after six interventions (*F* = 65.59, *p* < 0.001). These results suggested that spatial cognitive interventions significantly improved the spatial cognitive abilities of the experimental group, whereas the control group, which did not receive any intervention, showed no significant changes in spatial cognitive levels.

In summary, the MRT scores of the experimental group significantly increased post-intervention, demonstrating the effectiveness of spatial cognitive intervention. The control group showed no significant changes, further supporting the efficacy of the intervention in enhancing spatial cognitive levels. These results provide a solid foundation for further research on spatial cognitive interventions.

### 3.5 Analysis of SSQ scores before and after intervention

The changes in SSQ scores before and after the intervention for both the experimental and control groups are illustrated below. Before the intervention, the experimental group’s scores were as follows: Nausea (63.88 ± 43.47), Oculomotor (69.54 ± 29.15), Disorientation (130.73 ± 63.34), and Total (94.52 ± 44.37). After the intervention, the scores significantly decreased to: Nausea (26.55 ± 27.13), Oculomotor (38.89 ± 25.17), Disorientation (71.41 ± 46.42), and Total (48.62 ± 33.53).

In contrast, the control group’s pre-intervention scores were: Nausea (65.83 ± 24.35), Oculomotor (70.87 ± 37.48), Disorientation (128.76 ± 70.24), and Total (95.18 ± 43.34). Post-intervention, the scores showed less significant changes: Nausea (48.18 ± 26.35), Oculomotor (62.91 ± 38.34), Disorientation (116.23 ± 70.44), and Total (81.16 ± 44.70).

A paired samples *t*-test indicated that the experimental group experienced significant reductions in all SSQ scores after the intervention. Nausea (*t* = 5.55, *p* < 0.001), oculomotor (*t* = 6.76, *p* < 0.001), disorientation (*t* = 6.61, *p* < 0.001), and total SSQ scores (*t* = 7.58, *p* < 0.001) all significantly decreased, indicating that the spatial cognitive intervention significantly alleviated VIMS symptoms (Table 1).

**Table 1:** SSQ scores before and after intervention in the experimental group.

	*n*	Scores		*t*	*p*
		Before intervention	After intervention		
Nausea	23	63.88 ± 43.47	26.55 ± 27.13	5.55	<0.001
Oculomotor	23	69.54 ± 29.15	38.89 ± 25.17	6.76	<0.001
Disorientation	23	130.73 ± 63.34	71.41 ± 46.42	6.61	<0.001
Total scores	23	94.52 ± 44.37	48.62 ± 33.53	7.58	<0.001

In the control group, significant changes were observed only in nausea and total SSQ scores post-intervention. Specifically, nausea (*t* = 3.49, *p* < 0.01) and the total scores (*t* = 3.30, *p* < 0.01) showed some reduction, while changes in oculomotor (*t* = 1.62, *p* = 0.12) and disorientation (*t* = 1.79, *p* = 0.90) scores were not significant. This suggested that without spatial cognitive intervention, the control group’s VIMS symptoms improved only minimally (Table 2).

**Table 2:** SSQ scores before and after intervention in the control group.

	*n*	Scores		*t*	*p*
		Before intervention	After intervention		
Nausea	20	65.83 ± 24.35	48.18 ± 26.35	3.49	<0.01
Oculomotor	20	70.87 ± 37.48	62.91 ± 38.34	1.62	0.12
Disorientation	20	128.76 ± 70.24	116.23 ± 70.44	1.79	0.90
Total scores	20	95.18 ± 43.34	81.16 ± 44.70	3.30	<0.01

Independent samples t-tests were further conducted to analyze the post-intervention scores of the experimental and control groups in terms of nausea, oculomotor symptoms, disorientation, and total SSQ scores. The results indicated statistically significant differences between the two groups post-intervention: nausea (*t* = −2.64, *p* = 0.01), oculomotor symptoms (t = −2.46, *p* = 0.02), disorientation (*t* = −2.49, *p* = 0.02), and total SSQ scores (*t* = −2.72, *p* = 0.01). But prior to the intervention, there were no significant differences between the experimental and control groups across the four measured dimensions.

These findings highlighted the effectiveness of spatial cognitive interventions in significantly reducing VIMS symptoms, as evidenced by the marked differences between the experimental and control groups across all measured dimensions.

## 4 Discussion

This study explored the effects of spatial cognitive interventions on VIMS and spatial cognitive abilities. The results indicated no significant differences between the experimental and control groups in terms of nausea, oculomotor symptoms, disorientation, total SSQ scores, and spatial cognitive ability scores before the intervention, demonstrating the comparability of baseline data between the two groups. However, post-intervention, the experimental group showed significant improvements across all measures, confirming the effectiveness of spatial cognitive interventions in enhancing spatial cognitive abilities and alleviating VIMS symptoms.

Specifically, the experimental group exhibited a significant reduction in total SSQ scores and scores on all subscales post-intervention, with the nausea subscale score decreasing by 58.44%. This finding is consistent with previous research, validating the effectiveness of the nausea subscale as an indicator for assessing VIMS (Smyth et al., 2019). The control group also showed some improvement in nausea and total SSQ scores, which may be attributed to the adaptation effect induced by repeated visual stimulation (Guedry, 1998; Stroud et al., 2005; Pavlou et al., 2011).

Additionally, this study found that spatial cognitive interventions using VR equipment significantly improved participants’ spatial cognitive abilities. The experimental group demonstrated significant improvement after only three intervention sessions, suggesting that the intervention duration could be further shortened to enhance participants’ interest and motivation while reducing fatigue and boredom (Tseng and Yang, 2011; Slatenšek et al., 2021). However, for individuals with initially lower cognitive abilities or those involved in complex tasks, a longer intervention duration may be necessary to ensure skill mastery and practical application (Kim et al., 2014).

One limitation of this study is the lack of control over the adaptation period for both the experimental and control groups. The experimental group, having undergone VR training, may have had a longer adaptation period to the simulator, potentially influencing the results.

Future research should further investigate the application of spatial cognitive interventions across different populations and optimize intervention protocols to balance intervention duration and effectiveness for the best training outcomes. Additionally, given the differences in adaptation times between the experimental and control groups, future studies should better control for extraneous variables to enhance the reliability and validity of the research.

## Data availability statement

The raw data supporting the conclusions of this article will be made available by the authors, without undue reservation.

## Ethics statement

The studies involving humans were approved by the Ethics Review Committee of the PLA Air Force Medical Center [Air Force Special (Scientific Research) no. 2022-203-PJ01]. The studies were conducted in accordance with the local legislation and institutional requirements. The participants provided their written informed consent to participate in this study.

## Author contributions

FW: Conceptualization, Data curation, Formal analysis, Funding acquisition, Investigation, Methodology, Project administration, Resources, Software, Supervision, Validation, Visualization, Writing – original draft, Writing – review & editing. SP: Conceptualization, Data curation, Formal analysis, Funding acquisition, Investigation, Methodology, Project administration, Resources, Software, Supervision, Validation, Visualization, Writing – original draft, Writing – review & editing. X-wL: Writing – original draft, Writing – review & editing. J-mL: Writing – original draft, Writing – review & editing. C-jQ: Writing – original draft, Writing – review & editing. M-hJ: Writing – original draft, Writing – review & editing. Z-gJ: Conceptualization, Data curation, Formal analysis, Funding acquisition, Investigation, Methodology, Project administration, Resources, Software, Supervision, Validation, Visualization, Writing – original draft, Writing – review & editing. S-gY: Conceptualization, Data curation, Formal analysis, Funding acquisition, Investigation, Methodology, Project administration, Resources, Software, Supervision, Validation, Visualization, Writing – original draft, Writing – review & editing.
